# Acute and Chronic Cardiovascular Adverse Events in Patients with Acute Myeloid Leukemia: A Systematic Review

**DOI:** 10.3390/cancers17030541

**Published:** 2025-02-05

**Authors:** Konstantinos C. Siaravas, Amalia I. Moula, Ioannis S. Tzourtzos, Christos E. Ballas, Christos S. Katsouras

**Affiliations:** 1 Department of Cardiology, University Hospital of Ioannina, 45500 Ioannina, Greece; siaravaskon@gmail.com (K.C.S.); ioannistzourtz@gmail.com (I.S.T.); 2 Achilopouleio General Hospital of Volos, 38222 Volos, Greece; amaliamoula1@gmail.com; 3 Department of Thoracic Surgery, University Hospital of Ioannina, 45500 Ioannina, Greece; ballaschristos@gmail.com

**Keywords:** cardiovascular toxicities, cardiotoxicity, acute myeloid leukemia

## Abstract

**Background/Objectives:** Patients with acute myeloid leukemia (AML) have a higher propensity for adverse cardiovascular outcomes, primarily due to the toxic effects of chemotherapeutic agents. The purpose of this systematic review is to explore the association of acute myeloid leukemia treatment with adverse cardiovascular events. **Methods:** We systematically screened the literature for studies providing comparative data on cardiovascular toxicities in patients treated for acute myeloid leukemia. After the initial search, 3649 papers were screened and a final total number of 46 were included for the review process. **Results:** Common chemotherapeutic agents used in AML may cause cardiovascular (CV) toxicities. A plethora of pathophysiological mechanisms are incriminated for these effects. Drug combinations may increase the risk in a synergistic way. In addition, common mutations of AML, personal history of previous cardiovascular disease and impaired heart function carry an increased complication risk. Biomarkers, as well as multimodality imaging, may be used for the early detection of cardiovascular toxicities. **Conclusions:** Increased risks of CV toxicity and comorbidities are observed among AML patients. With all the available diagnostic modalities, early detection and CV prevention strategies can improve the patient’s prognosis and quality of life.

## Simple Summary

This paper examines the cardiovascular adverse events in patients with acute myeloid leukemia. Common chemotherapeutic drugs that are used for the treatment of acute myeloid leukemia are discussed, along with the potential pathogenetic mechanisms. Furthermore, the diagnostic modalities for the early detection of toxicity and the appropriate preventive strategies are presented. This systematic review aims to help the identification of common cardiac toxicities focusing on the appropriate early diagnosis and prevention of these adverse effects.

## 1. Introduction

Acute myeloid leukemia (AML) is a common hematologic malignancy. The prevalence of the disease is around 1% of all cancers overall and 10% of all new cases of hematologic cancer per year. There is a peak age of the disease around the age of 60 years, with over 55% of AML patients being above 65 years old. Furthermore, there is a male predominance of AML, with 4.5% per 100,000 males compared to 3% of females [1].

In AML, myeloid cells are arrested during differentiation, leading to the accumulation of immature cells, which in turn leads to ineffective erythropoiesis. The origin of the disease is in the bone marrow. Several genetic mutations and abnormalities in cytogenesis, as well as rearrangements within blast or immature cells, contribute to the disease [2].

Due to population aging and the better prognosis with cancer treatments, there is an increase in the incidence of cancer patients with coexisting cardiovascular diseases (CVDs) [3]. Many common risk factors such as hypertension, obesity, diabetes, sedentary lifestyle, dietary habits, socio-economic factors, socio-economic stress and smoking are important for the pathogenesis of those diseases [4].

Nowadays, there are treatment advancements and many options for patients with AML, such as chemotherapy with or without stem cell transplantation, radiation treatment and targeted therapies. Cardiovascular diseases have a high prevalence among AML patients [5]. Acute and long-term adverse events might occur because of the coexistence of two diseases or due to the potential cardiovascular toxicities of AML treatment. Studies have shown that patients with heart failure have a significantly increased incidence of hematologic cancers compared with patients without heart failure [6]. Further research is needed to define whether heart failure induces hematologic cancer or vice versa.

This current paper is a systematic review aiming to identify and describe acute and chronic cardiovascular disorders in patients with AML, the potential cardiotoxic side effects of common chemotherapeutic regimens and the AML-associated comorbidities that may also lead to CVD.

## 2. Materials and Methods

A systematic literature review was conducted in compliance with the updated Preferred Reporting Items for Systematic Reviews and Meta-Analyses (PRISMA) 2020 statement [7].

The PubMed database was searched until the end of October 2024. The following search terms were used: (acute myeloid leukemia) AND (cardiovascular disease OR cardiovascular toxicities OR cardiotoxicity). No additional filters were used during the literature search.

Inclusion criteria for the selection of studies for the current systematic review were systematic reviews, meta-analyses, original research or prospective studies, English publication language, and studies including data about acute myeloid leukemia and cardiovascular events or toxicities and the timing of the first CVD occurrence after the initial AML diagnosis. Exclusion criteria were case reports, non-English publication language and studies with the inclusion of the pediatric population (in this review, the pediatric population is defined as patients with AML aged < 16 years).

**Figure 1:**
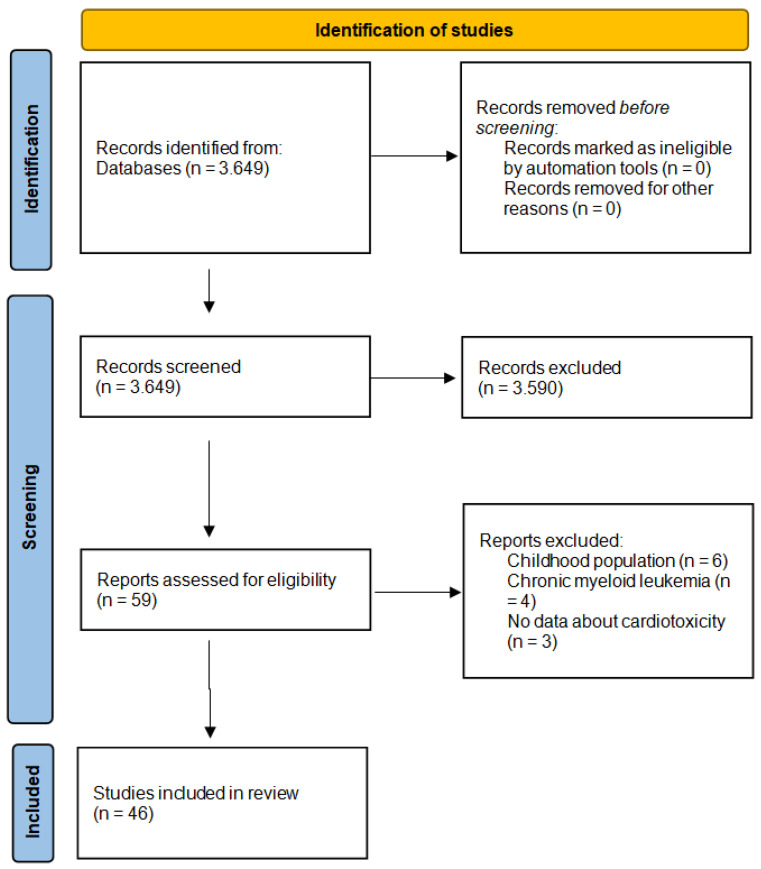
Flowchart of the selection of studies after a systematic review of the literature.

A total number of 3649 papers was screened, using the above-mentioned search terms. After the initial title screening, abstract and full-paper screenings were conducted in order to select the papers fulfilling the inclusion or exclusion criteria. After the exclusion of papers, a total of 46 studies were selected for the analysis. No duplicates needed to be excluded. Figure 1 depicts the flowchart of the selection of studies.

## 3. Acute Myeloid Leukemia and Cardiovascular Toxicities

### 3.1. AML and Cardiotoxicity

AML is a common hematologic malignancy. Chemotherapeutic regimens that are used in patients with leukemia have potential cardiotoxic effects. Anthracyclines, hypomethylating agents, cytarabine, CPX-351 (cytarabine with daunorubicin), gemtuzumab with ozogamicin, quizartinib, enasidenib, gilteritinib, ivosidenib, olutasidenib and venetoclax are the most commonly used [8]. Common CVDs that can be caused by AML drug regimens are heart failure, myocarditis, arrhythmias through QT segment prolongation, bradyarrhythmias, atrial fibrillation, pericardial disease, acute pericarditis and anthracycline-related cardiotoxicity [8]. Table 1 summarizes the common chemotherapeutic regimens used in AML, along with their cardiovascular toxicities and possible mechanisms of disease.

Clonal hematopoiesis of indeterminate potential (CHIP)-related mutations can be a precursor to hematologic malignancies and may be related to a raised risk of progression to AML. In addition, heart failure hospitalizations, worse outcomes and prognosis are related to CHIP mutations [13]. Patients with the DNMT3A mutation present with an increased incidence of heart failure as a comorbidity. This mutation is usually found in older individuals. Intensive chemotherapeutic drug combinations and high dosages can raise the risk of cardiotoxicity. TP53 and ASXL1 mutations raise the absolute risk of heart failure hospitalizations, ACS events, stroke and episodes of venous thromboembolic events (VTE) [2]. Furthermore, IDH 1/2 mutations increase the risk of coronary artery disease, valvular heart disease and cardiac dysfunction [14].

Other conditions that may be associated with AML are also responsible for the pathogenesis of cardiovascular disorders. For example, disseminated intravascular coagulation (DIC) is a common complication of patients with AML and has around a 10–20% prevalence in this population. DIC is associated with cardiovascular complications and can lead to ACS, CAD and heart failure [15].

Crosstalk between tumor cells and cardiomyocytes can lead to increased cardiotoxicity. The release of tumor cell-derived interleukin leads to cardiomyocyte dysfunction with detrimental effects. Metabolic substrates, energy metabolism pathways and cellular signaling pathways raise the susceptibility of myocardial cells to chemotherapy-related toxicity [16].

An increased incidence of 2–12% of venous thromboembolic events (VTEs), such as deep venous thrombosis (DVT) and pulmonary embolism (PE), has been noted among AML patients. Racial and age differences were also observed in the incidence of VTE among AML patients, with black males, female gender and older age being those with increased probability for having VTE episodes [17].

### 3.2. Anthracycline Cardiotoxicities

Anthracyclines are one of the most commonly used chemotherapeutic drug categories in patients with AML and one of the first-line regimens. There are well known cardiovascular adverse effects associated with their use. A reduction in left ventricular ejection fraction (LVEF) and the induction of heart failure are the commonest.

There are known predictors for this cardiotoxic effect, such as the dose of the treatment, presence of conventional cardiovascular risk factors, stage at the moment of diagnosis and patient sex. Pre-chemotherapy LVEF is an important factor, as patients with lower LVEF in the beginning of treatment advance to heart failure more frequently. Overall, previous history of heart failure, CAD and cardiomyopathies, as well as hypertension and chronic kidney disease (CKD), are potent risk factors of anthracycline toxicity [18]. The presence of a JAK mutation further increases the incidence of anthracycline cardiotoxicity.

The total cumulative dosage increases the relative risk of cardiovascular complications in cases of a cumulative dose of daunorubicin above 400 mg/m^2^. The reduction in LVEF after anthracycline usage may be a contraindication for patients who may need to proceed with further treatments, such as allogeneic stem cell transplantation [17].

There is a statistically significant correlation between the use of anthracyclines and the induction of cardiac arrhythmias. In addition, there is an increase in ST segment deviations and T wave inversions, heart rate, sinus tachycardia, conduction abnormalities and atrio-ventricular blocks. There are no differences in atrial and ventricular ectopic premature contractions, as well as in the incidence of atrial flutter among patients with a history of AML treated with anthracyclines. Higher doses of anthracyclines, especially above 200–300 mg/m^2^, seem to significantly increase the relative risk of arrhythmias [19].

Other studies have shown that parameters measured in simple blood tests and liver panels are also related to the cardiotoxic effects of anthracyclines. Total and direct bilirubin, total platelet count number and blood glucose values are independent factors for the presentation of CVD toxicity. Platelet count is a contributing factor of toxicity (especially the dynamic changes in platelet count during chemotherapy cycles), whereas bilirubin has a cardio-protective action [20].

Different pathophysiological mechanisms have been proposed for anthracycline-related left ventricular disease (ARLVD). Anthracyclines reduce the antioxidant capacities of cardiac cells by increasing oxidative stress with the production of reactive oxygen species (ROS). In addition, the production of alcoholic anthracycline metabolites interferes with the energy chain in the cellular metabolism pathways, leading to intracellular calcium concentration changes. Increasing cellular calcium levels can induce mechanisms of apoptosis and programmed cellular death [21].

As anthracyclines are first-line chemotherapeutic drugs that are used in patients with AML, and because of the increased risk of CVD toxicity during the treatment, there is increasing interest in the cardio-protective strategies for primary prevention. Chelation therapy with dexrazoxane in some cases has an accepted safety profile and reduces the relative risk of CVD by preventing, reducing or delaying cardio-toxic effects of patients treated with anthracyclines [22]. High anthracycline blood levels during infusions are also a potent factor for cardiac toxicity. The prolongation of anthracycline infusion time leads to a reduction in peak blood concentration and thus reduces the risk of adverse effects. The use of liposomal formulations and nanotechnologies, or use of the drug combination cytarabine with daunorubicin (CPX- 351), can lead to the prolongation of plasma drug half-time and to a reduction in the distribution of drug volumes in comparison with AML patients treated with non-liposomal forms of treatment [18]. Treatment with angiotensin-converting enzyme inhibitors (ACEi) or angiotensin receptor blockers (ARBs) and also beta-blockers may further reduce the potential toxicity of chemotherapy.

There is a wide timeline regarding to the onset of cardiotoxicity after chemotherapy with anthracyclines. Generally, it takes around 1 month after the beginning of chemotherapy until 6 months for more cases to manifest the cardiotoxic effects. Regarding the reversibility of toxicity complications of anthracyclines, there seem to be low rates of LVEF increase, even after starting treatment for heart failure. Approximately 70% of patients have a partially reversible LVEF and most other patients do not improve at all [23].

Acute promyelocytic leukemia (APL) is a subtype of AML characterized by a chromosomal translocation t (15; 17) (q22; q12–21), resulting in the fusion of the promyelocytic leukemia (PML) gene with the retinoic acid receptor alpha (RARα) gene. This fusion results in the disruption of the differentiation of myeloid cells, leading to leukemia. All-trans retinoic acid (ATRA), a vitamin A derivative, in combination with arsenic trioxide is an effective treatment for this condition [24]. Interestingly, ATRA, in contrast with other drugs for AML, may have a cardio-protective role, as it was shown to reduce the cardiotoxicity of anthracycline by regulating key genes of the RARG-TOP2B pathway [25].

### 3.3. Cytarabine Cardiotoxicities

Cytarabine alone or in combination with anthracyclines, as a part of the CPX-351 chemotherapeutic drug regimen, is commonly used as a first-line drug for the treatment of AML. In combination with anthracyclines, it can increase the incidence of heart failure due to its synergistic effect. When used alone, it is associated to angina pectoris, pericarditis and bradyarrhythmias [8]. Cytarabine competes with cytidine to incorporate itself into DNA and works as an antimetabolic agent that blocks the function of DNA polymerase, leading to the toxic effects of the drug in myocardial cells [8].

### 3.4. Venetoclax Cardiotoxicity in AML Patients

Venetoclax is a novel B-cell leukemia inhibitor drug that is used for the treatment of AML. Venetoclax use is associated with a higher incidence of CVD, especially when compared to anthracyclines. This may be caused because patients with AML that used venetoclax are older and have many cardiovascular risk factors. There is a tendency towards earlier onset of major adverse cardiovascular events (MACEs) in patients in whom venetoclax is used. Furthermore, there is increased concern because there are currently no studied cardio-protective treatments [9].

Several cardiotoxic effects of venetoclax have been proposed as potential mechanisms of adverse action. In rat models of myocardial cells, venetoclax increases the hypertrophic markers, β-Mhc and Bnp, and pro-apoptotic proteins such as Bax. In addition, it induces a reduction in the expression of an anti-apoptotic protein of Bcl-2. Furthermore, venetoclax increases the ROS and oxidative cellular environment, activates proinflammatory reactions with a decrease in Sod-2 protein level and increases gene and protein expression of Nf-kb-p 65 factor [9].

### 3.5. Hypomethylating Agents and Combinations

Hypomethylating agents (HMAs), alone and in combination with other chemotherapeutic drugs such as venetoclax, can have adverse CVD effects. Patients treated with Ven-HMA will develop MACE and especially non-ST segment elevation acute coronary syndromes (NSTE-ACSs), cardiomyopathies, heart failure and pericardial effusions. As mentioned earlier, CHIP mutations and polymorphisms, as well as older age and male sex, have a statistically significant increase in MACE prevalence [10].

The combination of venetoclax with hypomethylating agents (Ven-HMA) is a newer generation treatment in comparison to the traditional CPX-351 first-line therapy. The combination of these drugs further increases the incidence of cardiac toxicities, but the mechanisms involved are not yet well understood [25]. Hypomethylating agents alone can cause cardiovascular toxicities due to naive immune mechanisms of action with impairment of DNA repair, apoptosis signaling and angiogenesis [26].

### 3.6. Tyrosine Kinase Inhibitors in AML

Tyrosine kinase inhibitors (TKIs) are usually used as a second-line treatment in addition to a standard regimen of anthracycline or CPX-351 drug combination. Common complications of TKIs are hypertension, VTE events (DVT or PE), arrhythmias due to QT segment prolongation, atrial arrhythmias and atrial fibrillation. The addition of nilotinib to the classic 7 + 3 regimens of anthracyclines with cytarabine did not further increase the incidence of heart failure. The addition of nilotinib did not show statistically significant differences in terms of stroke, ACS, peripheral arterial disease, severe episodes of PE or VTE and arrhythmias. A QTc prolongation was higher in the nilotinib group of patients [11].

### 3.7. Gemtuzumab Ozogamicin Therapy and CVD

Gemtuzumab and ozogamicin (Mylotarg) treatment is a targeted treatment option in patients with AML experiencing a relapse. No serious cardiac side effects have been noted with the use of this drug combination [12].

### 3.8. Previous Cardiovascular Disease and AML Toxicity

AML and CVD are common comorbidities. In many cases, patients presenting with AML may have a previous history of CVD. One third of the AML population have at least one cardiovascular condition before the onset of AML treatment. Patients with a history of CVD, as well as a lower LVEF and depressed heart function, have lower rates of stem cell transplantation. History of CVD was associated with acute CVD complications, whereas patients without previous CVD have an increased relative risk of late-onset complications. Additionally, older age raises the baseline risk for developing cardiotoxicities.The number of CAD risk factors at the initiation of treatment further increases the risk of adverse cardiovascular effects [11].

In a meta-analysis of patients with a history of heart failure, there is a significantly increased risk of hematologic malignancies with a hazard ratio of 1.63 and a confidence interval of (1.15–2.33). The five studies that were used in this meta-analysis showed an increased risk of hematologic malignancies in patients with previous heart failure history. However, there were no specific data about the exact type of hematologic malignancy [5]. Only one of these studies showed an increased, non-significant risk of multiple myeloma among the other hematological malignancies in patients with heart failure. There were no specific data about AML in this patient population [27].

### 3.9. Acute Coronary Syndromes in AML Patients

AML patients may present with different ACS subtypes. AML patients have a lower prevalence of ST elevation myocardial infarctions (STEMIs) [28]. Increased rates of type II myocardial infarctions are a result of oxygen delivery/demand mismatch, the main causes of are anemia, dehydration, infections, sepsis and drug toxicities [27].

Several mechanisms are responsible for ACS presentation in AML patients. Systemic coagulopathy, platelet dysfunction and thrombocytopenia may be responsible for ACS. The overall numbers of platelets may be reduced; however, their activation is easier. This leads to cell aggregation to the coronary plaque, which in turn increases the risk of adverse effects [5]. Furthermore, coronary artery thrombosis may be a complication of AML and a cause of ACS [29]. Another possible mechanism described in some case reports is leukostasis due to hyperleukocytosis, which increases blood viscosity [30].

### 3.10. Percutaneous Coronary Interventions in AML Patients

**Figure 2:**
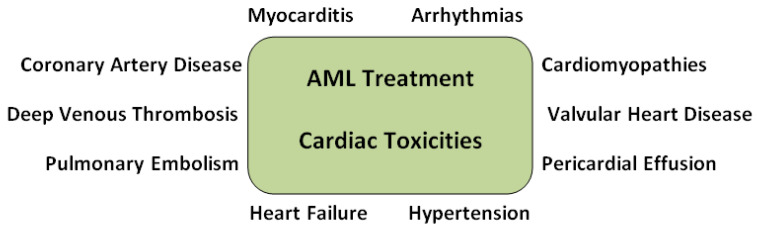
Cardiovascular toxicities caused by AML chemotherapy.

Patients with AML and a co-existing acute coronary syndrome are candidates for a revascularization procedure. Current guidelines suggest revascularization of the culprit lesion during the acute phase of an ACS. Percutaneous coronary intervention (PCI) is the most common procedure performed. AML patients have the highest mortality among hematologic malignancy patients and among the other leukemias. Acute leukemia has higher complication rates and AML has an exactly 5-fold increase in in-hospital mortality. In addition, AML has a statistically significant higher complication risk. In particular, a higher risk of bleeding and access site hematomas is noted after the revascularization procedure. Renal impairment and thrombocytopenia may also be considered in chemotherapy patients when making decisions about revascularization, as they increase complication rates [31]. Figure 2 summarizes the cardiovascular toxicities cause by AML chemotherapy.

### 3.11. Allogeneic Stem Cell Transplantation and CVD

During their treatment, AML patients may need to have an allogeneic stem cell transplantation. Cardiac dysfunction and previous cardiotoxicity may become contraindications for the decision to proceed with the procedure. Furthermore, CVD often leads to heart failure and poor performance status, which reduce the transplantation success. In addition, cardiovascular complications can occur during the follow-up period. Generally, they are categorized as early post-transplantation or late post-transplantation complications [32].

**Figure 3:**
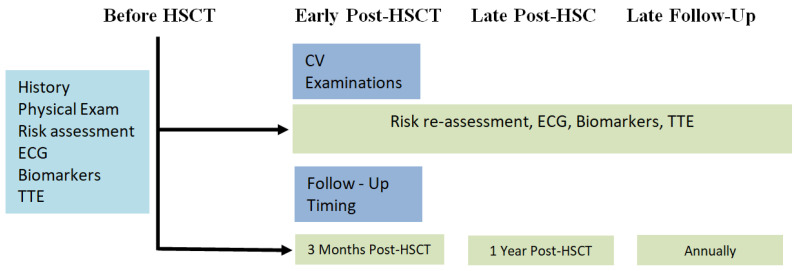
Follow-up of patients receiving a HSCT. Abbreviations: HSCT: hematopoietic stem cell transplantation, ECG: electrocardiogram, TTE: transthoracic echocardiography.

Early complications are usually myocarditis and heart failure, especially in patients with depressed LVEF before transplantation. Furthermore, coronary artery disease, ACS, pericarditis, pericardial effusions, tamponade and arrhythmias (more commonly atrial fibrillation) have been noted in the early period. Late complications include heart failure, premature CAD and stroke. Furthermore, many common CAD risk factors, such as new onset or worsening hypertension, exaggerated dyslipidemia or diabetes [32]. Complications may be influenced by the previous dosage and the combination of chemotherapeutic drugs used for the treatment of AML before remission. Anthracyclines are notorious for early and late post-transplantation heart failure and CV toxicities. The cumulative dose of drugs used increases the absolute risk of toxicity. More specifically, cumulative doses of daunorubicin above 500 mg/m^2^ raised the relative risk of heart failure by 14% in the 2-year period post-transplantation [33]. Figure 3 depicts the follow-up of patients receiving a HSCT.

### 3.12. Timeline of Cardiotoxicity

The potential cardiotoxic effects of chemotherapy may become apparent in different time intervals during the follow-up of AML patients. These effects are classified into three categories. Acute toxicity becomes apparent during or immediately following drug administration and is the less common type of toxicity. Sub-acute cardiomyopathy and heart failure occur usually 8 months to 1 year after the final dose administration. Late-onset toxicity occurs after the 1st year of completion of treatment, with a peak after 5–6 years of the last dosage administration. Late-onset toxicities are often progressive and irreversible [34].

There are no specific data available on the exact timeline of each cardiovascular toxicity of AML. Generally, as heart failure is the most prevalent toxicity, the above data are basically related to the timeline of heart failure after a cardiotoxic treatment. There is a lack of data about the exact time of CVD appearance after cardiotoxic AML treatments. Table 2 summarizes the timeline of CVD in AML patients after using cardiotoxic chemotherapeutic regimens.

### 3.13. Detecting Cardiotoxicity in AML Patients

The detection of cardiotoxic effects of chemotherapy is important for the further management of patients and for the improvement in outcomes. When toxicity is apparent and there is a low LVEF, it is easier to detect it, even by less experienced physicians. The baseline cardiovascular risk and the factors that increase susceptibility to cardiac toxicity are an important consideration before the initiation of potential cardiotoxic chemotherapeutic drugs. Patient follow-up is based on the measurement of cardiac biomarkers (troponins and natriuretic peptides) but also on imaging modalities, especially echocardiography and cardiac magnetic resonance (CMR). The exact follow-up schedule and the necessary blood and imaging tests are determined by the patient’s baseline risk of cardiac toxicity, as well as the therapeutic regimens and drug combinations used [35].

Generally, anthracycline-related left ventricular dysfunction is the most studied toxicity in AML patients. There are no specific data about the early toxicity and treatment of other chemotherapeutic agents. Regarding the use of biomarkers for myocardial injury and drug toxicity, there are no specific cardiac markers for the AML patient population. Troponins and natriuretic peptides are still the cornerstones of biomarker use in AML patients, similarly to cardiovascular toxicities caused by other cancer types. Patients with high and very high cardiovascular risk of potential cardiotoxic effects should have a measurement of baseline cardiac troponins and natriuretic peptides. Strict follow-up with a repeat measurement before every chemotherapy cycle during the first six cycles is mandatory, especially for early toxicity detection. After the completion of treatment, the above biomarkers should be measured again at 3 months and 1 year after the last treatment administration for the detection of sub-acute toxicities. For late toxicities, biomarker measurements should be repeated in the second, third and fifth year, and then every five years. Patients with low or moderate risk of potential cardiotoxicity may have a more lenient cardiology follow-up with biomarker measurements every second therapy cycle [35]. Other experimental potential biomarkers that are being tested in patients with hematologic malignancies are high-sensitivity CRP (Hs-CRP), interleukin-6 (IL-6), myeloperoxidase (MPO), fatty-acid-binding protein (FABP), glycogen-phosphorylase-binding protein (GPBP) and neuregulin-1 (NRG-1). The above biomarkers have not yet become a part of standard clinical practice [21].

There is increasing interest in the early detection of cardiotoxicity in patients treated with AML. The need for early detection springs from the worse outcome in patients in whom it is discovered late and from the fact that there is only partial reversibility when the LVEF is low. The novel echocardiography technique of speckle tracking imaging may detect early cardiotoxic events before a reduction in LVEF is apparent. The detection of such events leads to the increased use of cardio-protective drugs (e.g. ACEi and ARBs and beta-blockers) that seem to delay or prevent cardiotoxicity [36]. Strain imaging is useful in detecting cardiotoxicity after stem cell transplantation in patients who previously received chemotherapy. A deformation analysis can detect early subendocardial increases in the longitudinal strain, even from the first month after the transplantation. In addition, further subepicardial deformation, which is a parameter for further myocardial dysfunction, can be detected in the first 3 months. Early strain imaging abnormalities in the first month have a predictive value for a reduction in LVEF by the end of the follow-up [37].

**Figure 4:**
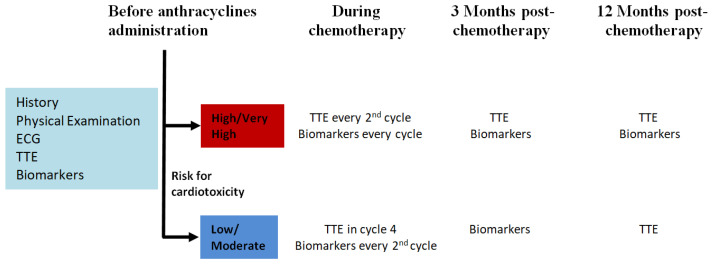
Timeline for the follow-up of high and very high-risk AML patients undergoing chemotherapy with anthracyclines. Abbreviations: ECG: electrocardiogram, TTE: transthoracic echocardiography.

The suggested echocardiographic intervals for the follow-up of patients receiving anthracyclines and at high risk of cardiotoxic adverse effects are as follows: during the baseline examination, then every second chemotherapy cycle, at 3 months and in the 1st year after the completion of treatment. In terms of biomarkers, after the year, it is suggested that TTE be repeated at the second, third and fifth year, or sooner if symptoms appear, and then every five years. Patients with hematopoietic stem cell transplantation (HSCT) need to have specific surveillance after the procedure and, especially for high-risk patients, biomarker (natriuretic peptide) and TTE evaluations are recommended at baseline, at 3 months and 1 year post-transplantation and then annually [35]. There is a lack of specific data about the use of CMR in AML patients with cardiotoxicity and the appropriate diagnostic usage. Thus, CMR should be used as in the case of non-AML patients following the general principles of heart failure diagnostics, until more data about AML become available. Figure 4 depicts the timeline for the follow-up of high and very high risk AML patients undergoing chemotherapy with anthracyclines.

Intima media thickness (IMT), ankle brachial index (ABI) and pulsed wave velocity in aorta (PWVAo) may be used in the early detection of peripheral arterial disease (PAD) as predictors of potential CAD and as potential markers of subclinical atherosclerosis [38].

### 3.14. Prevention and Treatment of Heart Failure in AML Patients

The general principles of the treatment of heart failure may be used in AML populations, especially when there is a reduction in LVEF. Little is known regarding the preventive strategies used for the cardiotoxic effects of chemotherapy in AML patients. Angiotensin-converting enzyme inhibitors delay the induction of anthracycline-related cardiotoxicity in AML patients [17]. One study investigated the effect of pretreatment with enalapril and carvedilol combination in acute leukemia patients for the prevention of chemotherapy-related cardiac toxicity. The study showed an increased ratio of preservation of LVEF and reduction in MACEs [39].

Furthermore, chelating treatment (i.e., dexrazoxane) reduces cardiotoxic effects and may help in the prevention of LVEF reduction in AML patients. In addition, a change in chemotherapeutic regimens (i.e. venetoclax–hypomethylating agents combination) may reduce the toxicity. The prolongation of anthracycline infusion time or use of lower suggested anthracyclines dosage can also be used as preventive strategies during chemotherapy [18].

In the clinical trials of heart failure, patients with malignancies and particularly with AML were excluded from the patient population. From the limited data that are available, cancer appears to be a major comorbidity in heart failure patients and a major cause of non-cardiovascular death. This fact became obvious because of the better prognosis of heart failure patients [40]. Regarding patients with solid tumors and hematologic malignancies, there are limited benefits for invasive cardiac procedures in advanced disease states. A multidisciplinary and patient-centered approach should be considered in order to obtain the most benefit from the cardiac interventions on cancer patients [41]. Finally, there are no specific data about the cardiac interventions and implantable devices in patients with AML.

## 4. Discussion

There are many common drug categories that are used for the treatment of AML and are related to adverse cardiovascular toxicities. There is great concern about the early detection of toxicities. Several cardio-protective strategies for preventing the toxicity of chemotherapeutic drugs are referred to above. Another potential drug combination that can be used, but that has only been tested in a small population of older patients with a personal history of previous cardiovascular disease, severely raising the risk of toxicities, was fludarabine, cytarabine and granulocyte colony stimulating factor (FLAG). The FLAG chemotherapy combination seems to be an effective and safe alternative to anthracycline use in certain subgroups of AML patients [42].

Because of the need for the early detection of cardiotoxicities and prevention of severe comorbidities and irreversible heart damage, there is a great interest in novel cardiac biomarkers apart from the classic troponins and natriuretic peptides. Glycogen phosphorylase BB enzyme has been used as an indicator of myocardial ischemia and necrosis. Patients with AML treated with anthracyclines were found to have increased levels of glycogen phosphorylase BB, which is suggested as a potential biomarker of CVD. At 6 months after treatment, elevated levels of glycogen phosphorylase BB have been correlated with diastolic left ventricular dysfunction [43]. An experimental rat model showed that treatment with venetoclax increased the levels of cardiac biomarkers, with a statistically significant increase in CK-MB levels [44]. On the other hand, there is a study with patients with hematologic malignancies and solid tumors, which presented postmortem histopathological findings and biomarker measurements. Patients were treated with antimetabolites, anthracyclines, targeted therapies and radiation treatment. Most patients in the hematologic malignancies population group were patients with AML. An AML subtype analysis showed no statistical difference in terms of cardiac troponins and CK-MB levels in these patients. On the contrary, higher BNP values were noted after the treatment in the follow-up of AML patients [45]. Further research is needed to clarify this conflicting evidence.

Risk scores are being tested for heart failure detection in AML patients treated with anthracyclines. The 21-point risk score is a simple six-parameter score that is being evaluated for potential heart failure complications. The parameters are AML, age above 60 years, history of pre-existing CVD, baseline GLS > −15%, LVEF <50% and anthracycline dose > 250 mg/m^2^. This score grades patients as being at low, moderate and high risk of heart failure. Scores corresponding to high-risk patients have been correlated with a higher cumulative incidence of MACE, heart failure and hospitalizations for CVD and lower chances of survival [46].

Although not included in the present review, AML is also a common hematologic malignancy in the pediatric population. Pediatric patients usually do not have a history of previous CVD or risk factors for CAD and, during the baseline assessment, are of low cardiovascular risk for cardiotoxicity. Young age (below 4 years), female sex, length of follow-up, Down syndrome and preparative regimens for stem cell transplantation (cyclophosphamide and total body irradiation) are prognostic factors for cardiotoxicity in the pediatric population. Toxicity can be early (within one year) from treatment initiation or late (after one year) [47]. Childhood AML has high remission rates and thus remission history and the total anthracycline cumulative dose are important risk factors for toxicities. A cumulative dose >250 mg/m^2^ is considered high in pediatric populations with AML. Amsacrine is another treatment that can be used and also may cause cardiovascular toxicities. Electro-cardiographic changes, atrial and ventricular arrhythmias, cardiomyopathies and heart failure can occur [48]. In addition, dexrazoxane has been used as a cardio-protective treatment in pediatric populations. Dexrazoxane was associated with a statistically significant reduction in cardiotoxic effects of anthracyclines in the pediatric population but this was limited to the outcomes that included subclinical cardiotoxicity [49].

Newer agents used for the treatment of heart failure, such as SGLT-2 inhibitors, have shown potential cardio-protective effects in cancer patients treated with anthracyclines. Specifically, empagliflozin showed a statistically reduced relative risk of detecting ARLVD in patients with breast cancer and diabetes or a history of heart failure with preserved ejection fraction [50]. More research needs to be carried out into the cardio-protective actions of SLGT-2 in certain other cancer types treated with anthracyclines and in patients with AML, as they interfere with many protective cellular mechanisms [51].

Trimetazidine is used for the treatment of symptomatic stable angina. The drug acts through inhibiting 3 ketoacyl coenzyme A thiolase, which decreases fatty acid oxidation. AML cells can survive the metabolic activation of fatty acid oxidation (FAO). FAO inhibitors have been developed and investigated for their therapeutic potential. Thus, trimetazidine and ranolazine (FAO inhibitors) can possibly target residual AML cells that survive chemotherapy-induced stress; in addition to their anti-anginal action in patients with coronary artery disease. More data are needed for patients with AML [52].

Nanotechnology and nanodelivery systems are created to yield an effective targeting of the therapeutic site of action of chemotherapy. Furthermore, delayed drug release and an extended drug half-life can lead to reduced potential cardiac toxicities in AML patients treated with anthracyclines. Doxil (PEGylated liposomal doxorubicine), Lipo-Dox (the next generation of Doxil) and DaunoXome are nanotechnologies that can improve daunorubicin (anthracyclines) pharmacokinetics and plasma chemical stability, leading to a reduction in the cumulative dosage needed. Similar nanomolecules are available for doxorubicin, pirarubicin and epirubicin [53].

Future research directions need to focus on the use of nanotechnologies to reduce the cardiac toxicities of chemotherapeutic agents. Furthermore, research can be carried out into the role of biomarkers in the early diagnosis of toxicities and patient follow-up. Potential new and more specific biomarkers should be studied. Preventive strategies may be sought with a prospective analysis in order to study their role in the outcomes or MACEs of AML patients. Finally, more specific diagnostic algorithms should be created and research into the use of multimodality imaging in cardiotoxicity detection in AML patients should be carried out.

## 5. Conclusions

AML is a common hematologic malignancy. The treatments used and the existing comorbidities may increase the incidence of CVD in AML patients. Cardiovascular toxicities may be acute or late and, mostly, they are related to chemotherapeutic regimens. Heart failure is the most common toxicity. Individual baseline risk evaluation can predict the probability of complications involving cardiac toxicity.

The proper early recognition of cardiac toxicities, before they lead to extensive structural and functional abnormalities, is extremely important. Several cardio-protective strategies, that include the dosage and combinations of chemotherapeutic agents; nanotechnologies; drug treatment for the prevention of toxicity, e.g., chelation therapy; and medications for reversing cardiac remodeling (i.e., ACEi, ARBs, B-blockers and SGLT-2 inhibitors), can be useful for reducing the probability of irreversible toxicities.

New technologies and the use of biomarkers and imaging may help in the early detection of potential toxicities. Natriuretic peptides and cardiac troponins are the most commonly used biomarkers, while echocardiography and CMR are used as the basic imaging modalities. All the above reduce adverse effects and improve patient outcomes and quality of life, leading to better prognoses with fewer complications for AML patients.

## Figures and Tables

**Table 1:** Cardiovascular adverse toxicities and mechanisms of commonly used chemotherapeutic agents in acute myeloid leukemia patients.

Chemotherapeutic Regimen	Cardiovascular Toxicity	Mechanisms
Anthracyclines [8]	Cardiomyopathy Heart failure Coronary artery disease Arrhythmias	Reactive oxygen species Energy chain metabolism Increased calcium levels Apoptosis Programmed cell death
Cytarabine [8]	Heart failure Angina Bradyarrhythmias Pericarditis	DNA polymerase dysfunction DNA production
Venetoclax [9]	Heart failure Coronary artery disease Atrial fibrillation	Increased hypertrophic markers Pro-apoptotic environment Oxidative stress Pro-inflammatory reactions
Hypomethylating Agents (HMAs) [10]	Heart failure Acute coronary syndromes Pericardial effusions	DNA repair Apoptosis Angiogenesis Immune reactions
Tyrosine Kinase Inhibitors [11]	Hypertension Venous thromboembolic events Ventricular arrhythmias Atrial fibrillation	Signal transduction Cellular processes
Gemtuzumab/Ozogamicin [12]	Hypotension Left ventricular dysfunction	Not specified

**Table 2:** Timeline of cardiovascular toxicities after use of chemotherapeutic regimens in patients with acute myeloid leukemia.

Type of Toxicity	Time from Chemotherapy Onset	Reversibility
Acute Cardiac Toxicity [34]	During or immediately following drug administration	Possibly reversible
Subacute Cardiac Toxicity [34]	Peaks in 3 months and can appear up to 8 months–1 year after the last dose administration	Possibly progressive and irreversible
Late Cardiac Toxicity [34]	After the first year of last dose administration, five or more years later	Possibly progressive and irreversible

## Abbreviations

The following abbreviations are used in this manuscript:

ARBs	Angiotensin receptor blockers
ARLVD	Anthracycline-related left ventricular disease
ACEi	Angiotensin-converting enzyme inhibitors
ACS	Acute coronary syndromes
AML	Acute myeloid leukemia
APL	Acute promyelocytic leukemia
CAD	Coronary artery disease
CHIP	Clonal hematopoiesis of indeterminate potential
CKD	Chronic kidney disease
CMR	Cardiac magnetic resonance
CVD	Cardiovascular disease
DVT FAO	Deep venous thrombosis Fatty acid oxidation
HMA	Hypomethylating agents
HSCT	Hematopoietic stem cell transplantation
IMT	Intima media thickness
LVEF	Left ventricular ejection fraction
MACE	Major adverse cardiovascular effects
NSTE-ACS	Non-ST elevation acute coronary syndrome
PCI	Percutaneous coronary intervention
PE	Pulmonary embolism
PML	Promyelocytic leukemia
ROS	Reactive oxygen species
SGLT-2 STEMI	Sodium Glucose Co-Transporter 2 ST elevation myocardial infarction
TKI	Tyrosine kinase inhibitors
VTE	Venus thromboembolic events
